# Evidence of a Protective Effect of Metformin on Abdominal Aortic Aneurysm Risk: Insights From an Observational Study and Mendelian Randomisation Analysis Using Putative Metformin Targets

**DOI:** 10.1111/ahg.70049

**Published:** 2026-07-23

**Authors:** Katie Louise Saxby, Svetlana Stoma, Frank Dudbridge, Nilesh J. Samani, Matthew J. Bown, Christopher P. Nelson

**Affiliations:** ^1^ Department of Cardiovascular Sciences University of Leicester Leicester UK; ^2^ NIHR Leicester Biomedical Research Centre University of Leicester Leicester UK; ^3^ Department of Population Health Sciences University of Leicester Leicester UK

**Keywords:** abdominal aortic aneurysm (AAA), Mendelian randomisation, metformin

## Abstract

**Aims:**

There is no proven treatment to prevent the growth or rupture of abdominal aortic aneurysm (AAA). As an aneurysm enlarges over time, the risk of fatal aortic rupture increases. Metformin, a drug usually prescribed to treat Type 2 diabetes, has previously been associated with reduced AAA risk in observational studies. Our aim was to assess whether there was a causal association between metformin treatment and AAA using Mendelian randomisation (MR).

**Methods:**

Logistic regression analysis was conducted in UK Biobank with 2972 AAA cases and 89,160 propensity‐matched controls. In addition, two‐sample MR analysis was performed, using a genetic proxy for metformin consisting of variants associated with both gene expression of seven metformin drug targets and decreased glycated haemoglobin (HbA1c) levels. Effect sizes for HbA1c were obtained from within UK Biobank, and for AAA risk from AAAgen, a multi‐ancestry meta‐GWAS analysis of 39,221 cases and 1,086,107 controls.

**Results:**

We found evidence of a protective association between self‐reported metformin treatment and reduced AAA risk in the observational analysis, OR 0.49 (95% CI: 0.41–0.59, *p* = 1.5 × 10^−14^). MR results support this finding, with an estimated decrease in AAA risk of 43%, OR = 0.57 (95% CI: 0.38–0.88, *p* = 0.010) per one standard deviation (sd) decrease in HbA1c via metformin gene targets, equivalent to the effect of a prescribed dose of metformin. This effect is specific to metformin target genes and was not seen using a general untargeted instrument.

**Conclusion:**

Our observational study found evidence that metformin use reduces the risk of developing AAA. The MR results support this finding, providing evidence that the association may be causal. Clinical trials are warranted to assess the efficacy of metformin to reduce the risk of aneurysm growth and rupture in people with AAA.

**Abbreviations**
AAA: abdominal aortic aneurysm
ADCY1: adenylyl cyclase
AMPK: AMP‐activated protein kinase
FBP1: fructose bisphosphatase‐1
GCG/GLP‐1: glucagon/glucagon‐like peptide‐1
GDF‐15: growth differentiation factor 15
HbA1c: glycated haemoglobin
MCI: mitochondrial complex I
MG3: mitochondrial glycerol 3
MR: Mendelian randomisation

## 1 Introduction

Abdominal aortic aneurysm (AAA) is clinically defined as a dilatation of the abdominal aorta measuring 30 mm or more (Wanhainen et al. 2019). The global prevalence is estimated to be 0.92% in adults aged 30–79 (Song et al. 2023) and is most prevalent in men over the age of 65 (Roychowdhury et al. 2023). Untreated, AAAs slowly grow, increasing the risk of rupture as they get bigger (Ibrahim et al. 2021). The mortality rate of patients with ruptured AAA is estimated to be 65%–85% (Roychowdhury et al. 2023). The only current treatment for AAA is surgical repair, which is itself associated with significant morbidity and mortality (Wanhainen et al. 2019). For people with small AAA, where the aneurysm size is below recommended surgical thresholds, there are no treatments available to slow AAA growth and prevent the need for surgery in the future. Furthermore, there are no treatment options for patients who are frail and not fit enough for surgery.

Diabetes is associated with reduced AAA risk (Aune et al. 2018) as well as reduced AAA progression or growth rate (Gellatly et al. 2024). Observational studies have shown that metformin, a commonly prescribed oral hypoglycaemic agent used commonly for the treatment of diabetes, is associated with a reduction in AAA incidence and reduced growth (Golledge et al. 2017) and may be a driver of the reduced risk seen in people with diabetes.

Our hypothesis was that metformin use is associated with reduced AAA disease risk. We tested this association both observationally, using UK Biobank, and via Mendelian randomisation (MR). MR utilises genetic variation as a natural experiment to investigate evidence of a causal association between a risk factor and an outcome (Davies et al. 2018). It is a popular method that can overcome a major limitation of observational studies, confounding, through the use of genetic instruments (Davies et al. 2018) (concept and assumptions described in the Supporting Information).

The metformin instrument was developed by researchers who applied the instrument in two publications. The first investigated an association between metformin and Alzheimer's disease via five known metformin target pathways and found statistically significant evidence of a protective effect in a general population (OR 0.85 [95% CI: 0.78–0.93]) and a population without diabetes (OR 0.96 [95% CI: 0.95–0.98]) (Zheng et al. 2022). This instrument was later updated to include two additional metformin target pathways when investigating the effects of metformin on cardiometabolic traits and diseases (Zheng et al. 2023). The authors report a 38% reduction in risk of coronary artery disease (CAD) for a standard deviation (sd) reduction in metformin‐targeted HbA1c (OR 0.62, 95% CI: 0.46–0.84), as well as reductions in body mass index (BMI), systolic blood pressure (SBP) and diastolic blood pressure (DBP) levels. Given the significant genetic correlation between CAD and AAA (Roychowdhury et al. 2023), and that high blood pressure and BMI are known risk factors for AAA, we hypothesised a consistent protective effect for AAA.

## 2 Methods

### 2.1 Observational Study

Logistic regression was used to investigate the effect of metformin use on AAA risk in participants from UK Biobank a large prospective cohort study consisting of over 500,000 individuals with extensive information from various resources, including self‐reported questionnaires and integrated healthcare records. Cases with AAA were identified solely using appropriate International Classification of Diseases (ICD) and OPCS Classification of Interventions and Procedures (OPCS‐4) codes linked to electronic medical records (Table S1a), identifying participants diagnosed with treated for or with AAA listed as a primary or secondary cause of death and the respective date this occurred.

ICD codes were also used to exclude unsuitable controls (participants with related conditions, e.g., thoracic abdominal aneurysm (Table S1b)).

Self‐reported medication field 20003 was used to identify participants that had declared taking metformin, anti‐hypertensives, lipid‐lowering drugs or other antidiabetic medication (Tables S2a and S2d). UK Biobank reports that this field was completed by 363,607 participants at baseline, with smaller numbers at follow‐up visits (Table S2e). Where individuals had no record of medication, it is assumed to be not prescribed. Metformin use needed to be reported at least 3 months prior to the date of AAA case assignment to be eligible, ensuring that our analysis investigated the effect of metformin on (future) disease risk.

Propensity matching was used to select a control group from eligible UK Biobank participants to ensure a good representation of the case group (Supporting Information).

Logistic regression was used to test the effect between metformin and AAA risk, adjusting for self‐reported lipid‐lowering medication and anti‐hypertensive medication as recorded in UK Biobank fields 20003, 6153 or 6177. In secondary analyses, diabetes status (defined using ICD codes or as self‐reported, Table S3) was also adjusted for in the model. Additional analysis was conducted where participants reported individual antidiabetic medication therapy, such as metformin only. This contrasted the main analysis where participants reported metformin use but may also have reported another antidiabetic medication such as sulfonylurea or insulin. As positive controls, individual use of insulin, sulfonylurea or thiazolidinedione was also analysed against AAA risk. As a negative control, the effect of metformin use on ‘handedness’ was tested (Supporting Information). To complete the analyses, diabetes status was also investigated for association with AAA (Supporting Information).

UK Biobank has approval from the North West Multi‐centre Research Ethics Committee as a research tissue bank.

### 2.2 Mendelian Randomisation

#### 2.2.1 Genetic Instrument to Proxy Metformin Action

We conducted MR using published genetic instruments for a collection of known metformin target pathways (Zheng et al. 2023). The genetic instruments were built using a two‐stage approach. First, a literature search was conducted to find known metformin drug targets. Genes known to associate with these targets were subsequently identified using CHEMBL (Gaulton et al. 2012) and Drug Bank (Drugbank Online 2024). Variants were then selected if found to be significantly associated with gene expression for any of these genes within a QTL study (Emilsson et al. 2018; Folkersen et al. 2017; GTEx Consortium 2020; Suhre et al. 2017; Sun et al. 2018; Võsa et al. 2021; Yao et al. 2018). Second, any variants retained in the first stage were then selected if they were also significantly associated with HbA1c levels in Europeans. This resulted in one instrument for each of seven pathways: AMP‐activated protein kinase (AMPK), growth differentiation factor 15 (GDF15), mitochondrial complex I (MCI), mitochondrial glycerol 3 (MG3), glucagon (GCG)/glucagon‐like peptide‐1 (GLP1), fructose bisphosphatase‐1 (FBP1) and adenylyl cyclase (ADCY1) consisting of a total of 34 variants near 25 genes. The instruments represent metformin's HbA1c‐lowering effect.

Effect sizes used within the MR were independently estimated in European‐ancestry participants within UK Biobank (Neale Lab 2018) (*n* = 344,182) using *Z*‐standardised HbA1c. Effect sizes represent sd changes in HbA1c where a unit change in the UK Biobank general population is estimated as 1.09% (∼6.75 mmol) (Zheng et al. 2022) (Table S4). This effect size is consistent with the observed effect estimated in a systematic review of metformin monotherapy in diabetic patients: 1.12% (95% CI: 0.92–1.32) (Hirst et al. 2012).

The metformin effect on AAA risk was also tested using a metformin instrument weighted using only unrelated participants of European ancestry without Type 2 diabetes (*n* = 338,425) (Zheng et al. 2022) (Table S5). Effect sizes also represent an sd change in HbA1c, estimated to be 0.62% in this non‐diabetic population (Zheng et al. 2023).

As a negative control, MR analysis was conducted to test evidence of association between metformin and right‐handedness (Supporting Information). MR was also used to assess evidence of association between insulin, sulfonylurea and thiazolidinedione antidiabetic medications and AAA (Supporting Information) using identified target genes (Qin et al. 2023).

#### 2.2.2 AAA Outcome

Effect sizes and standard error estimates for AAA risk were obtained from AAAGen, the largest genetic study on AAA risk to date: a multi‐ancestry (European and African) meta‐GWAS comprising 39,214 cases and 1,086,107 controls (Roychowdhury et al. 2023). There was a small amount of sample overlap between the exposure and outcome, as the HbA1c effect sizes are estimated within UK Biobank and AAAGen includes 1041 UK Biobank AAA cases and up to 22,049 controls from UK Biobank as part of the meta‐analysis. Although potential bias from this overlap was anticipated to be minimal, an AAAgen analysis excluding UK Biobank was utilised for sensitivity analysis (Table S6).

Ethical approval was not required for the two‐sample MR study as publicly available summary statistics were used.

#### 2.2.3 Statistical Analysis

MR was conducted using the inverse variance weighted (MR–IVW) method (Burgess et al. 2019) as our primary analysis for each individual target pathway. We also considered the general metformin effect using an instrument, including all seven targets combined.

We assessed weak instrument bias using the *F*‐statistic (Burgess, Dudbridge, et al. 2016) and performed sensitivity analysis excluding variants from the combined instrument where the variant was estimated to be weak (*F*‐statistic < 10). To assess whether individual variant drug targets were driving or influencing the effect seen in the combined target, we further removed variants from targets where there was only a single variant in the instrument (ACDY1, GDF‐15, GCG, FBP1 and MG3), leaving MCI and AMPK. The Steiger test for directionality (Hemani et al. 2017) was used to investigate the causal direction of effect of each variant, and MR analysis was applied on variants surviving Steiger filtering.

The adopted instruments are designed to assess the impact of a change in HbA1c due to specific metformin target pathways. We investigated the targeted specificity of the instrument (in contrast to a general HbA1c effect) by using a genome‐wide HbA1c instrument sourced from the same paper (Zheng et al. 2022) on our outcome, AAA risk. An instrument was also prepared to test for association between Type 2 diabetes and AAA using a recent multi‐ancestry GWAS (Mahajan et al. 2022) to verify that the previously reported null effect (Ibrahim et al. 2021) was consistent when tested using the latest exposure and outcome data. The metformin effect on AAA risk was also tested using the instrument derived from a non‐diabetic population.

We applied several sensitivity analyses to test and satisfy the key MR assumptions. To assess potential weak instrument bias or pleiotropy, we applied the weighted‐median (Bowden et al. 2015), weighted‐mode (Hartwig et al. 2017), MR‐RAPS (Zhao et al. 2020) and MR‐PRESSO (Burgess et al. 2019) to the combined instrument and tested the intercept in MR‐Egger (Burgess et al. 2019). Effect estimates were compared across methods for evidence of consistency.

All analyses were conducted with TwoSampleMR v0.5.6 in R v4.3.1.

## 3 Results

### 3.1 Observational Study

#### 3.1.1 Cohort Description and Baseline Characteristics

Of 502,398 UK Biobank participants, 3023 AAA cases were identified, and 497,410 suitable controls, after removal of 1965 participants with related conditions. A total of 27 participants first reported taking metformin after the recorded date of AAA diagnosis and were excluded from the analysis. Prior to propensity score matching, a further 2931 participants were excluded (24 cases, 2907 controls) with undetermined smoking status.

Propensity matching was used to identify suitable controls for the remaining 2972 AAA cases. Table 1 shows the baseline characteristics of the resulting case/control cohort as well as for all UK Biobank participants for comparison. Propensity matching successfully balanced age between AAA cases and controls, with the AAA cases having a mean baseline age of 63.9 (sd = 4.6) and controls 64.0 (sd = 4.9), significantly older than seen across all UK Biobank participants (mean 57.0, sd = 8.1). A suitable number of male controls were identified from propensity matching, ensuring that the proportion was consistent across the two groups (83.6% males in cases and 85.8% males in controls), compared to 45.6% across all participants. Results of matching on smoking status led to an improvement in balance between cases and controls, although the proportion of current smokers in cases (31.4%) remained higher than in controls (21.2%), though substantially higher than the general UK Biobank population (10.5%). Matching on previous smokers was more successful, with 50.1% of cases reporting previous smoker and 56.1% of controls, compared with 34.4% of all UK Biobank participants.

**TABLE 1:** Baseline characteristics by abdominal aortic aneurysm (AAA) case, propensity‐matched controls and all UK Biobank participants.

Variable		AAA cases *n* = 2972		Matched controls *n* = 89,160		All UK Biobank *n* = 502,398	
		Mean/Count	Standard deviation/%	Mean/Count	Standard deviation/%	Mean/Count	Standard deviation/%
Age		63.9	4.6	64.0	4.9	57.0	8.1
Male		2485	83.6	76,474	85.8	229,079	45.6
Smoking status							
	Current	932	31.4	18,871	21.2	52,962	10.5
	Previous	1490	50.1	50,011	56.1	173,021	34.4
	Never	550	18.5	20,278	22.7	273,467	54.4
	Prefer not to say	—	—	—	—	2057	0.4
	Missing	—	—	—	—	891	0.2
Medication							
	Metformin	128	4.3	5031	5.5	14,650	2.9
	Other antidiabetic	108	3.6	3891	4.4	12,299	2.4
	Anti‐hypertensive	1323	44.5	27,665	31.0	93,123	18.5
	Lipid‐lowering	1541	51.9	31,003	34.8	94,685	12.9
BMI		27.8	4.3	28.6	4.5	27.4	4.8
SBP		146.1	19.5	145.9	20.0	139.7	19.7
DBP		83.2	10.6	83.8	11.5	82.2	10.7
LDL‐C		3.4	0.9	3.3	1.0	3.6	0.9
Angina		832	28.0	11,856	13.3	34,186	6.8
CAD		1465	49.3	21,134	23.7	58,953	11.7
Diabetes		674	22.7	14,173	15.9	47,819	9.5
Hypertension		2709	91.2	72,288	81.1	326,882	65.1
Stroke		466	15.7	7232	8.1	22,057	4.4

*Note*: Smoking status and biomarker measures sourced at baseline. Medication data obtained from self‐reported UK Biobank fields 20,003, 6153 or 6177 (any single instance reported at baseline or follow‐up). Metformin, antidiabetic, lipid‐lowering and anti‐hypertensive medications were identified using British National Formulary (BNF) codes and cross‐referenced to UK Biobank field 20,003. A full list of UK Biobank drug codes is provided in Tables S2a and S2d. Disease status obtained from ICD and OPCS4 codes. Table S2f includes information on the first instance that participants reported metformin use.

Abbreviations: BMI, body mass index; CAD, coronary artery disease; DBP, diastolic blood pressure; SBP, systolic blood pressure.

Propensity matching by age, sex and smoking status to identify suitable controls has also resulted in balancing other baseline characteristics associated with AAA between the case and control groups, and so biomarkers and medication usage that are elevated in the AAA case group are also elevated in the matched controls relative to the overall UK Biobank population (Supporting Information). A total of 128 of the participants in the AAA case group self‐reported metformin use (4.3%), higher than the overall UK Biobank population (2.9%), with the highest level seen in the control group (5.5%). This is also seen for diabetes status, with 22.7% of AAA cases having been diagnosed and 15.9% of controls, compared with 9.5% of the whole UK Biobank population.

#### 3.1.2 Results of Regression Analysis

**FIGURE 1:**
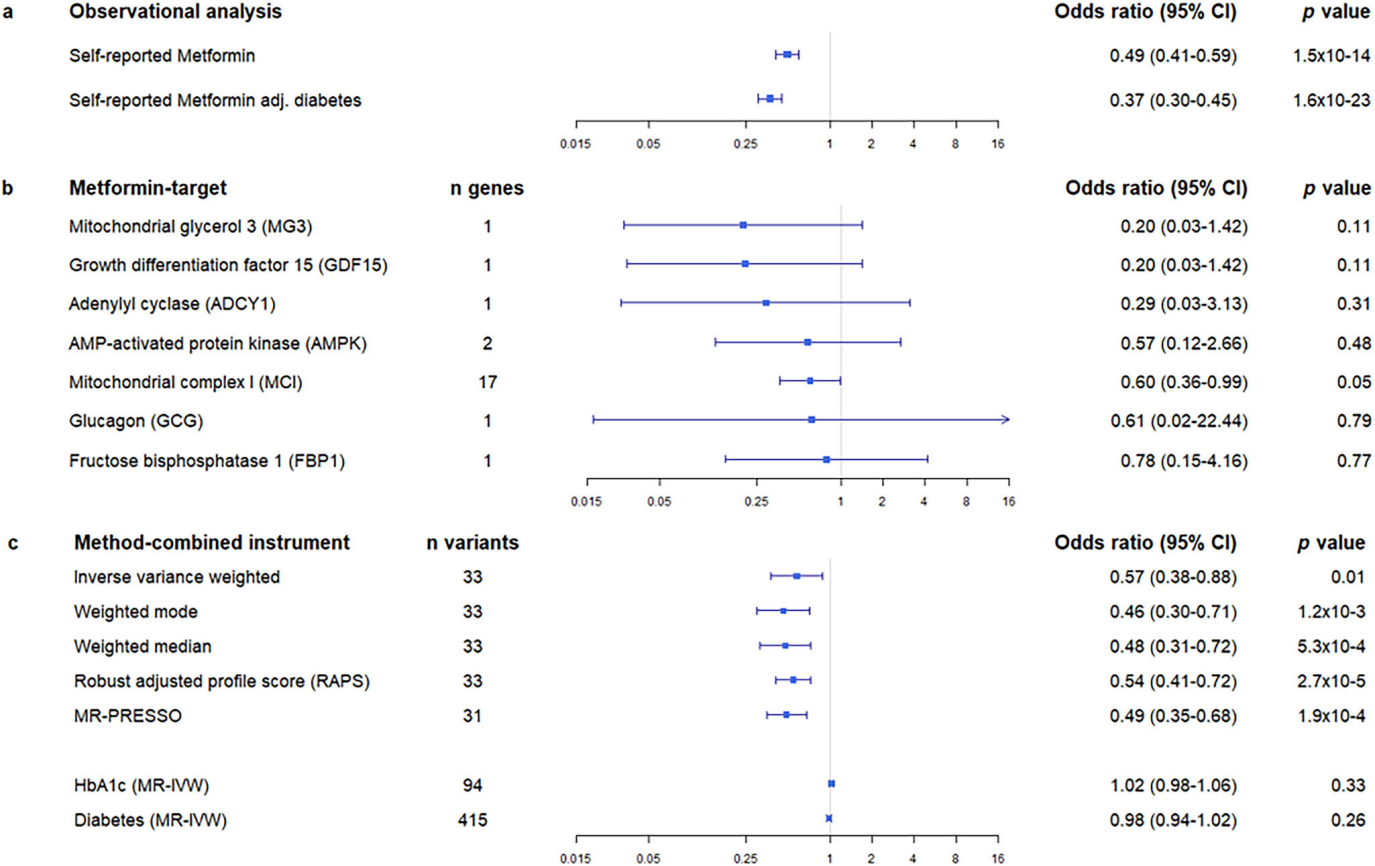
**Effects of metformin reduction on AAA risk, observationally and using genetically proxied HbA1c reduction via metformin target pathways on AAA risk. Panel a**: Estimated association between self‐reported metformin use and AAA risk in UK Biobank participants. Adjusted for lipid‐lowering medications and anti‐hypertensives. Secondary analysis further adjusted for diabetes status. **Panel b**: Genetic proxying of metformin use on genetically predicted HbA1c levels via seven known metformin target pathways in a general population estimated using MR‐IVW, showing OR and 95% confidence intervals. ‘*n* genes’ indicates the number of genes in each pathway. **Panel c**: sensitivity analysis of the combined 33 variant instrument (the MR‐PRESSO estimate represents IVW after the removal of two potential outlier variants), a genome‐wide HbA1c estimate using inverse weighted variance method, and a diabetes instrument. ‘*n* variants’ indicates the number of variants in each analysis. For MR analyses, the odds ratio estimates for metformin represent a 1.09% decrease (∼6.75 mmol/mol or 1 sd) in HbA1c.

Logistic regression was used to estimate the protective effect of metformin on AAA risk in the propensity‐matched cohort of 2972 cases and 89,160 controls. After adjusting for self‐reported lipid‐lowering and anti‐hypertensive medications, metformin was estimated to reduce AAA risk with a point estimate of 51% with compatible estimates ranging from 40% to 59% (OR 0.489, 95% CI: 0.407–0.587) (Figure 1, Panel a). Additional analysis adjusting for diabetes status estimated a greater protective effect of metformin (0.369, 95% CI: 0.303–0.448). No evidence of association was found between metformin use and handedness (Supporting Information).

The ‘metformin only’ analysis results were also consistent with the primary analysis (Table S7b). Sample sizes were small for other antidiabetic medications, so results should be interpreted with caution, but a protective effect was also seen for ‘insulin only’ use (Table S7a).

### 3.2 Results of MR

To understand if the putative metformin targets express in aortic artery tissue, a multi‐gene query was run including all 25 gene targets within GTEx (The Genotype‐Tissue Expression (GTEx) Project) and found that 24 (excepting GCG) are significantly expressed in aortic artery tissue (Figure S1). Of the 34 independent genetic variants (*r* ^2^ < 0.001), we found 33 available within AAAgen summary statistics (rs3136476 not found). Analysing each target pathway separately revealed consistent estimated protective effects against AAA risk (Figure 1, Panel b) with no evidence of heterogeneity (*I* ^2^ = 0%, Cochran's *Q* test 2.58, *p* value 0.859). The combined instrument of 33 variants found statistically significant evidence of association between HbA1c‐lowering variants via known metformin targets and the outcome, AAA risk: OR = 0.57 (95% CI: 0.38–0.88), indicating potential evidence of a causal association. The combined instrument estimates a 43% reduction in risk due to a 1.09% decrease in HbA1c in the UK Biobank general population. Results filtered after applying the Steiger test for directionality resulted in one variant being excluded (Table S8) and the estimated OR marginally affected: OR 0.62 (95% CI: 0.42–0.92) (Table S9). MR analysis using a genome‐wide HbA1c instrument of 94 variants showed no evidence of a causal association with AAA risk, OR = 1.02 (95% CI: 0.98–1.06), consistent with the HbA1c effect being specifically driven via metformin target pathways. Similarly, an MR using a Type‐2 diabetes instrument of 415 variants found no evidence of a causal association with AAA risk, OR = 0.98 (95% CI: 0.94–1.02), confirming results of the null effect seen in a previous study (Ibrahim et al. 2021).

As there was some sample overlap of 2%, we estimated the potential bias to have altered the log odds by 0.004 (Burgess, Davies, et al. 2016). Removing the UK Biobank cohort from AAAgen and recalculating the MR did not materially alter the results (Figure S2). We also note a potential for increased heterogeneity between the datasets due to mixed ancestry in AAAgen, although allele frequencies between included samples were found to be consistent (Roychowdhury et al. 2023).

All sensitivity analyses were applied to the combined instrument as it shows strong consistency in effect size and direction across all target pathways (Figure 1, Panel b), and the number of genes is limited within each individual target pathway. The MR‐Egger intercept point estimate was very close to zero with compatible ranges of −0.019 and 0.007 (−0.006, 95% CI: −0.019 to 0.007, *p* value = 0.39) suggesting no evidence of directional pleiotropy with respect to decreasing HbA1c.

Protective effects were estimated using the combined instrument prepared in a non‐diabetic population (Figure S3, Panel a) with an estimated risk reduction of 21% but with compatible values ranging from 4% to 34%, OR = 0.79 (95% CI: 0.66–0.96) per sd change (0.62%). Effect sizes were again seen to be consistent in sensitivity analyses (Figure S3, Panel b).

The primary instrument weighted using all participants of European ancestry from UK Biobank was found to have only a single target with an *F*‐statistic < 10 (GCG), but sensitivity analysis to assess bias found no evidence of significant bias as exclusion of GCG did not affect the results (Table S10). The same conclusion was made when analysing only multi‐variant drug targets (AMPK and MCI). Two weak instruments were observed in the non‐diabetic population instrument (GDF‐15 and AMPK), but again, sensitivity analysis found no impact on study conclusions (Table S10). Results of the negative control and other antidiabetic medications can be found in the Supporting Information with supporting evidence in Tables S10 and S13 and Figure S4.

## 4 Discussion

This study used both observational and MR analyses to test for evidence of association between metformin use and AAA risk. The results of the observational analysis provide evidence of a substantial protective effect, a finding that is corroborated in the MR analysis indicating that the association is potentially causal. The secondary regression analysis demonstrates that the association is not due to diabetes status.

MR analysis used genetic instruments prepared in a non‐diabetic and general population to assess the potential therapeutic benefit of metformin in reducing AAA risk via known metformin targets in a general population. The results provide potentially causal evidence that metformin may reduce AAA risk via an HbA1c‐lowering mechanism specific to targeted pathways. These results provide a potential understanding of the mechanisms that may drive observational study findings. Further research of each individual target may lead to identification of other potential drug repurposing suitable for use in reducing AAA risk and potentially slowing growth. Results were shown to be robust and consistent across all targets, populations and in sensitivity analyses, with attenuated but still significant effect sizes also seen using the instrument constructed in a non‐diabetic population.

It is known that the magnitude of effect estimates from MR studies should be interpreted with caution as they represent the lifetime protective effect, here acting as a proxy for metformin use via targeted HbA1c‐lowering variants, and yet in this study, the results of the observational analysis indicate that the effect size is plausible. The design of the observational study, using self‐reported metformin use in UK Biobank, with the majority of participants reporting this at baseline assessment (Table S2e), has meant that largely long‐term metformin use on AAA risk has been assessed. This may explain why the estimated protective effects are of similar magnitude between the observational and MR results.

The reduction in HbA1c that achieves the estimated risk reduction in the MR study in the UK Biobank general population is within the estimated pharmacological effect of a clinical metformin dose seen in clinical trials of diabetic patients, providing further evidence to support that the estimated effect size is viable. The HbA1c‐lowering effect of metformin in non‐diabetic populations is not well studied, making it difficult to speculate whether this effect size is achievable in a general population, providing further justification of the need for clinical trials. Although we have not shown a direct effect on AAA progression, the inferred benefit of metformin treatment provides additional justification for ongoing clinical trials (Golledge et al. 2021; Lareyre and Raffort 2021). HbA1c is itself involved in many biological pathways, and critically, we found no evidence of a causal association between a genome‐wide HbA1c instrument and AAA risk, giving further evidence to the targeted specificity of an HbA1c change in the metformin target pathways.

We acknowledge that the results of the observational analysis may be impacted by confounding, as well as that the vast majority of patients taking metformin will do so due to having diabetes, and it may not be appropriate to generalise these results to individuals without diabetes; however, the findings of the MR analysis based on a sample of participants without diabetes suggest the metformin effect may be generalisable. Assumptions have been made on medication status for metformin, anti‐hypertensive, lipid‐lowering and other‐diabetic medication. It is likely that some of the participants have missing data for these medications along with an element of uncertainty in participants self‐reporting taking medication, due to inaccuracies in patient recall. The analysis was limited with little follow‐up medication data and, therefore, did not allow for changing therapies over time or for multiple antidiabetic medications. Our secondary analysis, restricted to participants reporting single antidiabetic medication therapy, was consistent with the results of our main metformin analysis.

Participants with linked prescription records could have been used and perhaps offered greater accuracy, but due to a large loss of sample size (these data are currently available for less than half UK Biobank participants), it was felt that the larger number of participants with self‐reported information and potentially longer term metformin use added greater value than fewer records that might offer a greater level of accuracy.

Our secondary analyses on insulin, sulfonylurea and thiazolidinedione use certainly provide motive for further work. Small numbers of each individual medication used in the observational study make the findings unreliable, and the mix of harmful and protective effects seen in the MR results also warrants investigation. Exploration of the mechanisms of each individual antidiabetic drug treatment to try and rationalise these findings would be valuable.

The validity of the instrument and its suitability to represent metformin effects in the MR analysis are potentially uncertain (Anderson and Williams 2023; Burgess et al. 2023), although the robust findings and high consistency in the estimates provide some reassurance of the reliability of the findings.

There may be other metformin pathways that have not been captured in the MR instrument, and as such, we do not present these findings as the full metformin treatment effect. Other pathways may counteract these protective effects and nullify or, even worse, cause harm and increase AAA risk, but given the results of the observational study, this appears unlikely. Findings from randomised clinical trial evidence have shown that metformin is associated with a modest reduction in BMI in obese non‐diabetic patients (Haber et al. 2024), consistent with the protective effect seen using the same metformin instrument in MR (Zheng et al. 2023). This consistency adds further reassurance that the instrument represents a genuine metformin action.

We have conducted these analyses using the best metformin instrument available, and our finding is entirely consistent with our observational study and also with other observational studies on AAA risk and AAA growth. For example, an observational study conducted using 1.2 million patients with diabetes in Taiwan found that those taking metformin were at a reduced risk of developing an aortic aneurysm, though this study was not specific to AAA (Hsu et al. 2016). Despite the consistency in evidence, we recognise that this article is limited to reporting results on seven known target pathways and that further work is required in order to ascertain the full estimate of a complete metformin effect.

We also note that the instrument was constructed on postulated downstream effects of metformin on glycaemic levels via known metformin targets, and we cannot evaluate the effect of metformin therapy in clinical practice (Burgess et al. 2023).

## 5 Conclusion

Our findings show a robust significant association between metformin and reduced AAA risk, with evidence that this may be due to lowered HbA1c levels via specific metformin target pathways. This finding indicates evidence of a causal association supporting the study of metformin use in a general population and in clinical trials currently in progress to assess the impact on AAA growth and risk of serious outcome (Golledge et al. 2021; Lareyre and Raffort 2021). These future and ongoing studies will soon help shed light on whether these targets represent a valid, full metformin effect or if the benefits could be cancelled out by pathways that we are currently unable to account for. With no medication currently available to slow disease progression or indeed reduce the risk of AAA, we remain hopeful that our findings, in alignment with those seen in observational studies, will confirm that metformin is a viable treatment option.

## Author Contributions

Study design: Katie Louise Saxby and Christopher P. Nelson. Data collection: Katie Louise Saxby and Svetlana Stoma. Data analysis: Katie Louise Saxby. Manuscript preparation: Katie Louise Saxby, Christopher P. Nelson, Matthew J. Bown, Frank Dudbridge, Svetlana Stoma and Nilesh J. Samani. Supervision: Matthew J. Bown, Christopher P. Nelson and Frank Dudbridge.

## Funding

This work was supported by the Wellcome Trust [222959/Z/21/Z] to K.L.S.; the British Heart Foundation Leicester Centre of Research Excellence [RE/34/130031]; British Heart Foundation [CD/14/2/30841 and RG/18/10/33842 to M.J.B., F.D. and C.P.N.]; Medical Research Council [MR/S037055/1 to F.D.] and British Heart Foundation [CH/F/22/90014] to M.J.B. For the purpose of open access, the author has applied a CC BY public copyright licence to any Author Accepted Manuscript version arising from this submission. This is a summary of independent research funded by the Wellcome Trust and the British Heart Foundation carried out at the National Institute for Health and Care Research (NIHR) Leicester Biomedical Research Centre (BRC). The views expressed are those of the author(s) and not necessarily those of the funders, the NIHR or the Department of Health and Social Care.

## Conflicts of Interest

The authors declare no conflicts of interest.

## Supporting information




**Supplementary Tables**: ahg70049‐sup‐0001‐TableS1–S13.xlsx

**Supplementary Figures**: ahg70049‐sup‐0002‐FigureS1–S4.docx

## Data Availability

Data relating to the Mendelian randomisation analysis are available in the article and in its Tables S4:S6, S8, S10 and S13. Data for the observational study was sourced from UK Biobank (Sudlow et al. 2015) under project 6077. Data access should be sought from UK Biobank.
