# Supplementary material for: Evidence of a Protective Effect of Metformin on Abdominal Aortic Aneurysm Risk: Insights From an Observational Study and Mendelian Randomisation Analysis Using Putative Metformin Targets
Source: Ann Hum Genet. 2026 Jul 23;90(5):294–302. doi: 10.1111/ahg.70049 (PMC13460526; doi:10.1111/ahg.70049)
Supplement: Supplementary file 1 [file AHG-90-294-s002.docx]

**Supplementary Material to: Evidence of a protective effect of Metformin on abdominal aortic aneurysm risk: Insights from an observational study and Mendelian randomisation analysis using putative Metformin targets**

**Contents**

| Supplementary Introduction: |  |
| --- | --- |
| Concept and assumptions of Mendelian randomisation studies | Page 2 |
|  |  |
| Supplementary Methods: |  |
| Selecting suitable controls using propensity matching | Page 2 |
| Observational analysis investigating evidence of association between Metformin and right handedness. | Page 2 |
| Mendelian randomisation analysis investigating evidence of a causal relationship between Metformin and right handedness. | Page 2 |
| Logistic regression of exclusive use of metformin, insulin, sulfonylurea and thiazolidinediones, and diabetes on AAA risk. | Page 3 |
| Mendelian randomisation analysis investigating evidence of a causal relationship between insulin, sulfonylureas, and thiazolidinediones on AAA risk. | Page 3 |
|  |  |
|  |  |
| Supplementary Results: |  |
| Balance of biomarkers, medication status and disease between cases and propensity matched controls | Page 4 |
| Observational analysis investigating evidence of association between Metformin and right handedness. | Page 4 |
| Mendelian randomisation analysis investigating evidence of a causal relationship between Metformin and right handedness | Page 4 |
| Logistic regression of other antidiabetics medication on AAA risk. | Page 4 |
| Results of Mendelian randomisation analysis of sulfonylureas, and thiazolidinediones on AAA risk. | Page 5 |
|  |  |
| Supplementary Figures: |  |
| Figure S1 - Multi-gene query of tissue specific gene expression of genes in Metformin instrument in GTEx. | Page 6 |
| **Figure S2** - MR associations between genetically predicted HbA1c levels and AAA using AAAgen samples excluding UK Biobank**.** | Page 7 |
| **Figure S3** - Associations between genetically predicted HbA1c levels in a non-diabetic UK Biobank population. | Page 8 |
| Figure S4 - Multi-gene query of tissue specific gene expression of genes in other antidiabetic medication instruments in GTEx. | Page 9 |

# Supplementary introduction

## Concept and assumptions of Mendelian randomisation studies

To illustrate MR, consider a single allele selected based on its strong association with the exposure, here, lowered levels of glycated haemoglobin (HbA1c) via Metformin targets. The effect of the allele on the outcome, AAA disease risk, is also estimated. If the allele has a protective effect on the outcome, this may imply that the exposure and the outcome have a causal association under certain assumptions, i.e., lowered HbA1c via Metformin targets causes a reduction in AAA risk. In practice we commonly use a group of genetic variants as our genetic instrument. For a genetic instrument to be valid, the following three assumptions must be met: 1) the instrument is associated with the exposure, 2), it is not associated with confounders of the exposure and outcome, and 3) it only affects the outcome via the exposure (Burgess et al., 2019).

# Supplementary methods

## Selecting suitable controls using propensity matching

Using the remaining UK Biobank population may have resulted in imbalance in confounding factors between baseline characteristics, as UK Biobank is known to be representative of a healthy population. The R-package ‘MatchIt’(Ho, D., Imai, K., King, G., Stuart. E, 2011) was used to propensity match 30 controls to every case. Matching was performed to minimise selection bias using nearest neighbour matching based on age (with a 2-year calliper), sex (exact), and smoking status (exact) at baseline (self-reported questionnaire), as individuals with AAA are likely to be older than the average UK Biobank participant, more likely to be male, and more likely to be a current or ex- smoker. Participants with missing or undeclared data on baseline smoking status were also excluded. Matching multiple controls to each case was to ensure sufficient numbers of Metformin users were captured in the cohort.

## Observational analysis investigating evidence of association between Metformin and right handedness.

Logistic regression was used to investigate the effect of ‘handedness’ using UK Biobank self-reported field (1707). Righthandedness was treated as case with all other categories set to control. Metformin status included any instance of self-reported Metformin use.

## Mendelian randomisation analysis investigating evidence of a causal relationship between Metformin and right handedness.

Right handedness was included as a negative control outcome, as it is a neurodevelopmental trait established early in life and is not known to be influenced by diabetes or vascular pathology.

GWAS summary statistics for right handedness (both sexes) were downloaded <https://www.nealelab.is/uk-biobank> (Neale, 2018) and variants in the Metformin instrument extracted.

## Logistic regression of exclusive use of metformin, insulin, sulfonylurea and thiazolidinediones, and diabetes on AAA risk:

Logistic regression was used to investigate the effect of individual antidiabetic medications using UK Biobank self-reported medication field (20002). Antidiabetic medications were grouped into categories: metformin, insulin, sulfonylureas and thiazolidinediones (Table S2d). Participants that reported exclusive use of antidiabetic medication were then identified. Consistent with the primary analysis, acceptable medication use was dependent on the drug being prescribed at least 90 days prior to the date of AAA classification. Logistic regression was used to investigate the effect of exclusive antidiabetic use separately, adjusting for antihypertensive and lipid-lowering drugs as per the main analysis.

Logistic regression was also conducted using diabetes status (identified using ICD, OPCS and self-reported questionnaire data (Table S3)), also with the requirement that the diabetes diagnosis was confirmed before the AAA diagnosis.

## Mendelian randomisation analysis investigating evidence of a causal relationship between insulin, sulfonylureas, and thiazolidinediones on AAA risk.

Target genes and locations of antidiabetic drugs investigated in the observation study (insulin, sulfonylureas, and thiazolidinediones) were sourced (Qin, 2023). Authors cross-referenced DrugBank and ChEMBL to identify encoding genes of target proteins and their locations.

To prepare a genetic instrument for the antidiabetic effect of these drugs on HbA1c, variants in these gene locations (+/- 1500 kb) were extracted from UK Biobank GWAS summary statistics of HbA1c levels (Neale Lab, 2018). Variants with a false discovery rate > 0.05 were excluded. Clumping was applied in a region of 10,000 base pairs with r^2^ of 0.001 ref panel, package to select almost independent variants as genetic instruments. Further analysis was conducted relaxing this threshold to r^2^=0.01.

Mendelian randomisation analysis methods were consistent with the primary analysis in the manuscript.

# Supplementary Results

## Balance of biomarkers, medication status and disease between cases and propensity matched controls

Baseline systolic blood pressure (SBP) is substantially higher in participants in the selected cohort with a mean value of 146.1 mmHg (s.d. 19.5) in AAA cases, 145.9mmHg (s.d. 20.0) in controls, compared with 139.7mmHg (s.d.=19.7) in the overall UK Biobank population. These higher levels are despite a larger proportion of AAA cases reporting anti-hypertensive medication at baseline (44.5% in AAA cases, 31.0% in AAA controls, and 18.5% overall). This effect is also seen with the proportion of participants with hypertension (identified from ICD codes and based on more recent information from HES data), at 91.2% of AAA cases, 81.1% of AAA controls, much higher than 65.1% of overall participants.

For LDL-C the AAA cases group (mean 3.4mmol/L, s.d=0.9) has the highest proportion of participants taking lipid-lowering medication (51.9%), whilst AAA controls have a marginally lower average LDL-C of of 3.3mmol (s.d.=1.0), with fewer participants (34.8%) reporting lipid-lowering medication. The average LDL-C for all participants was slightly higher with a mean of 3.6mmol (s.d.=0.9), but with far fewer participants taking lipid-lowering medication (12.9%).

Elevated numbers of participants with diseases associated with AAA were seen in the AAA case group such as angina, stroke and coronary artery disease (CAD) (28.0%, 15.7% and 49.3% of participants respectively), significantly higher than the levels in all UK Biobank participants (6.8%, 4.4%, and 11.7%). Higher levels were also seen in the matched controls compared to the overall group but not to the same extent as for the cases (13.1%, 8.1% and 23.7%).

## Observational analysis investigating evidence of association between Metformin and right handedness.

81,225 participants indicated right-handedness with 4,504 reporting Metformin use (5.5%). 10,289 of participants that were classified as ‘other handedness’ with 618 reporting Metformin use (5.7%). There was no evidence of association between Metformin use and handedness OR 0.977 (0.896-1.066).

## Mendelian randomisation analysis investigating evidence of a causal relationship between Metformin and right handedness

The variants included in the Metformin instrument were identified in the GWAS summary statistics for righthandedness (both sexes) and harmonised with the Metformin instrument (Table S11). No evidence of association was found (OR 1.013, 95%CI 0.985, 1.042, p-value 0.352) (Table S12). Sensitivity analysis provided consistent estimates of a null effect.

## Logistic regression of exclusive use of metformin, insulin, sulfonylurea and thiazolidinediones, and diabetes on AAA risk

Logistic regression was used to investigate evidence of association of individual antidiabetic therapy (from self-reported questionnaire data in UK Biobank) and AAA disease risk.

2,463 individuals (58 AAA cases (2%), 2405 controls (2.7%)) in the AAA case and propensity matched controls reported ‘Metformin only’. Sample sizes for exclusive use of the other antidiabetic medications were small: 745 reported insulin only (8 cases (0.3%), 737 controls (0.8%)), 339 reported sulfonylureas only (15 cases (0.5%), 324 controls (0.4%)), and 55 reported thiazolidinediones only (3 cases (0.1%), 52 controls (0.1%)) (Table S7a).

Logistic regression was also used to validate the association between diabetes status and AAA. 14,616 individuals were identified with diabetes (443 AAA cases (16.2%), and 14,173 controls (15.9%)) within the AAA cases and propensity matched controls (Table S7a).

The findings from the logistic regression based on Metformin use (exclusive of any other anti-diabetic medication) showed a protective effect: OR 0.488 (95%CI 0.371-0.630, p-value 1.0x10^-7^), consistent with the findings of our main analysis that examined participants self-reporting Metformin use (but included participants that had reported other antidiabetic medication use) (Table S7b**)**. Insulin use was also found to have strong protective effect on AAA risk (OR 0.221, 95%CI 0.101-0.415, p-value 2.2x10^-5^) (Table S7b**)** but these findings were based on a small sample size.

No evidence of association was seen between sulfonylureas (OR:0.982, 95%CI 0.558-1.593, p-value 0.95), or thiazolidinediones (OR: 1.130, 95% CI 0.275-3.074, p-value 0.84) and AAA risk (Table S7b), but this analysis was limited to very small sample sizes and is not conclusive. A protective effect of diabetes status was seen (OR 0.734, 95%CI 0.659-0.817, p-value=1.8E-08) (Table S7b).

## Mendelian randomisation analysis investigating evidence of a causal relationship between insulin, sulfonylureas, and thiazolidinediones on AAA risk.

Genetic instruments were prepared to represent the HbA1c lowering effect of three antidiabetic medications: insulin, sulfonylureas and thiazolidinediones. Variants within the gene regions +/- 1500kb window were extracted from UK Biobank summary statistics (Neale,Lab) (Table S13a). Instruments that were clumped using a strict r^2^ of 0.001 resulted in a single variant instrument for insulin (F-statistic 18.4), and two variants per instrument representing sulfonylureas (F-statistic 47.8) and thiazolidinediones (F-statistic 107.7) (Table S13b). Using a less stringent clumping threshold of r^2^ of 0.01 resulted in genetic instruments for insulin, sulfonylureas and thiazolidinediones with two, three and five variants, with F-statistics 13.4, 35.9 and 50.9 respectively (Table S13c).

To understand if the targets of these antidiabetic medications express in aortic artery tissue, a multi-gene query was run within GTEX (The Genotype-Tissue Expression (GTEx) Project) and found that insulin was most highly expressed, and moderate expression from PPARG (thiazolidinediones) and KCNJ11 (sulfonylureas), but there was no evidence of expression in the second sulfonylureas gene (ABCC8) Figure S4.

The genetic proxy for insulin estimated a protective effect of similar magnitude to the observational study, but there was no evidence to support a causal association: OR 0.242, 95%CI 0.046-1.267, p-value 0.093 (Table S13d). Sulfonylureas were estimated to have a harmful effect, such that a standard deviation decrease in HbA1c via the sulfonylureas target (via ABCC8) was associated with an increase in risk of 475% (OR 5.745, 95%CI (2.612-12.639), p-value 1.38x10^-5^). A protective effect of 43% was estimated using the thiazolidinediones proxy: OR 0.573, 95%CI 0.346-0.948, p-value 0.03) (Table S13d).

The point estimate of the effect of insulin on AAA risk using the two variant genetic proxy was a protective with an OR of 0.365 but with compatible estimates ranging from 0.086 to 1.541. (OR 0.365, 95%CI 0.086-1.541, p-value 0.170) (Table S13e). The sulfonylureas instrument estimated an increase in 365% (OR 4.654, 95%CI 1.702-12.724, p-value 0.003) of AAA risk for a standard deviation decrease in HbA1c, slightly attenuated compared to the single instrument findings. The results of the 5 variant thiazolidinediones instrument was consistent with the 2-variant analysis; a protective effect of 42% (OR 0.578, 95%CI 0.365-0.916, p-value 0.02).

**Supplementary Figures**

**Figure S1.** Multi-gene query of tissue specific gene expression in GTEx. All but one of the genes (GCG) selected for being involved in the action of seven known Metformin drug-targets have evidence of expression in aorta tissue (AMPK -	PRKAG2, PRKAA1, PRKAG2, MCI – NDUF*, MG3 – GPD2).

**Figure S2. MR associations between genetically predicted HbA1c levels and AAA using AAAgen samples excluding UK Biobank.** Where n genes indicates the number of genes in each pathway. Odds ratio (OR) estimates represent a 1.09% decrease (~6.75mmol/mol or 1 s.d.) in HbA1c. **Panel a:** Primary analysis of individual Metformin-drug target pathways estimated using MR-IVW, combined pathways of 33 genetic variants in the instrument, diabetes instrument, and genome-wide HbA1c estimate using inverse weighted variance method showing OR and 95% confidence intervals. **Panel b:** Sensitivity analysis of the combined 33 variant instrument. The MR-PRESSO estimate represents IVW after the removal of two potential outlier variants.

**Figure S3** Associations between genetically predicted HbA1c levels in a non-diabetic population via 24 genes/ 33 variants and abdominal aortic aneurysm risk from two-sample Mendelian randomisation analyses. Where n genes indicates the number of genes in each pathway. Odds ratio (OR) estimates represent a 0.62% decrease (1 s.d.) in HbA1c in the non-diabetic population. Panel a: Primary analysis of individual Metformin-drug target pathways estimated using MR-IVW, combined pathways of 33 genetic variants in the instrument, diabetes instrument, and genome-wide HbA1c estimate using inverse weighted variance method showing OR and 95% confidence intervals. Panel b: Sensitivity analysis of the combined 33 variant instrument. The MR-PRESSO estimate represents IVW after the removal of two potential outlier variants.

**Figure S4.** **Multi-gene query of tissue specific gene expression in GTEx.** All but one of the genes (ABCC8 - Sulfonylurea) selected have evidence of expression in aorta tissue, with insulin having the greatest levels (INSR - Insulin, PPARG - Thiazolidinediones, KCNJ11 & ABCC8 - Sulfonylureas).
